# The value of whole-body MRI instead of only brain MRI in addition to 18 F-FDG PET/CT in the staging of advanced non-small-cell lung cancer

**DOI:** 10.1186/s40644-025-00852-6

**Published:** 2025-03-11

**Authors:** Hanna Holmstrand, M Lindskog, A Sundin, T Hansen

**Affiliations:** 1 Radiology, Department of Surgical Sciences, Uppsala University, Uppsala University Hospital, entry 70, 1st floor, Uppsala, 751 85 Sweden; 2 Department of Immunology, Genetics and Pathology, Uppsala University, Dag Hammarskjölds väg 20, Uppsala, 751 85 Sweden; 3 Department of Pelvic Cancer, Section of Genitourinary Oncology, Karolinska University Hospital, Eugeniavägen 3, Solna, 171 76 Sweden

**Keywords:** Non-Small-Cell lung carcinoma, Magnetic resonance imaging, Positron emission tomography computed tomography

## Abstract

**Background:**

Non-small cell lung cancer (NSCLC) is a common neoplasm with poor prognosis in advanced stages. The clinical work-up in patients with locally advanced NSCLC mostly includes ^18^F-fluorodeoxyglucose positron emission tomography computed tomography (^18^F-FDG PET/CT) because of its high sensitivity for malignant lesion detection; however, specificity is lower. Diverging results exist whether whole-body MRI (WB-MRI) improves the staging accuracy in advanced lung cancer. Considering WB-MRI being a more time-consuming examination compared to brain MRI, it is important to establish whether or not additional value is found in detecting and characterizing malignant lesions. The purpose of this study is to investigate the value of additional whole-body magnetic resonance imaging, instead of only brain MRI, together with ^18^F-FDG PET/CT in staging patients with advanced NSCLC planned for curative treatment.

**Material and methods:**

In a prospective single center study, 28 patients with NSCLC stage 3 or oligometastatic disease were enrolled. In addition to ^18^F-FDG PET/CT, they underwent WB-MRI including the thorax, abdomen, spine, pelvis, and contrast-enhanced examination of the brain and liver. ^18^F-FDG PET/CT and WB-MRI were separately evaluated by two blinded readers, followed by consensus reading in which the likelihood of malignancy was assessed in detected lesions. Imaging and clinical follow-up for at least 12 months was used as reference standard. Statistical analyses included Fischer’s exact test and Clopped-Pearson.

**Results:**

28 patients (mean age ± SD 70.5 ± 8.4 years, 19 women) were enrolled. WB-MRI and FDG-PET/CT both showed maximum sensitivity and specificity for primary tumor diagnosis and similar sensitivity (*p* = 1.00) and specificity (*p* = 0.70) for detection of distant metastases. For diagnosis of lymph node metastases, WB-MRI showed lower sensitivity, 0.65 (95% CI: 0.38–0.86) than FDG-PET/CT, 1.00 (95% CI: 0.80-1.00) (*p* < 0.05), but similar specificity (*p* = 0.59).

**Conclusions:**

WB-MRI in conjunction with ^18^F-FDG PET/CT provides no additional value over MRI of the brain only, in staging patients with advanced NSCLC.

**Trial registration:**

Registered locally and approved by the Uppsala University Hospital committee, registration number ASMR020.

**Supplementary Information:**

The online version contains supplementary material available at 10.1186/s40644-025-00852-6.

## Background

Lung cancer is the second most commonly diagnosed cancer worldwide and the leading cause of cancer-related death [1]. It is histologically broadly divided into non-small-cell lung cancer (NSCLC), representing 80–85% f patients, and small-cell lung cancer (SCLC) [2]. Because of absent or unspecific symptoms in early stages, the majority of lung cancer patients are diagnosed with advanced disease [3]. Locally advanced disease (stage 3 in the TNM 8th edition classification) includes mediastinal lymph node metastases and/or primary tumor invading adjacent structures such as the chest wall, but without signs of distant metastases [4].

Provided a good performance status, these patients are treated with curative intention using chemoradiotherapy (CRT) with consolidative immune checkpoint inhibition [5]. Unfortunately, the cancer recurs in the majority of stage 3 patients and in a subset of about one third early after CRT, indicating that distant metastases may have remained undetected during routine diagnostic workup. At least 20% of NSCLC patients present with oligometastatic disease [6], commonly defined as 1–5 lesions [7]. Improved long-term survival has been shown following multimodal treatment, typically CRT or surgery in combination with stereotactic ablative radiation therapy (SABR) [8]. Therefore, it is paramount to detect all macroscopic metastatic lesions at first NSCLC diagnosis.

The clinical work-up in lung cancer staging often includes a ^18^F-fluorodeoxyglucose positron emission tomography/computed tomography (^18^F-FDG-PET/CT) because of its superior sensitivity in comparison to computed tomography (CT) alone [9]. However, interpretation of ^18^F-FDG-PET/CT may be challenging, especially regarding small lesions and lymph nodes, because focal ^18^F-FDG-uptake is not specific for malignant lesions [10].

This limitation has been addressed with studies of alternative PET tracers, such as 3’-deoxy-3’-fluorothymidine (FLT), with possibly higher specificity than 18F-FDG[11]. Another approach would be to use an additional morphological method, such as whole-body magnetic resonance imaging (WB-MRI), to facilitate characterization of unequivocal lesions. Previous studies have found diverging results reporting on one hand higher specificity for WB-MRI than for ^18^F-FDG PET/CT in the detection of pulmonary masses [12], mediastinal lymph node metastases [13, 14] and bone metastases [15] and on the other hand comparable results for T, N and M staging [16–18]. MRI of the brain is superior to ^18^F-FDG-PET/CT for detection of brain metastases, which are common in advanced stages of NSCLC [19, 20].

The aim of this study was to evaluate the added value of performing WB-MRI instead of merely MRI of the brain, in addition to ^18^F-FDG PET/CT in advanced stages of NSCLC.

## Methods

This prospective study was approved by the regional ethics committee (EPN: 2016/326). All procedures performed were in accordance with the 1964 Helsinki declaration or comparable ethical standards. Written informed consent was obtained from all participants.

### Study population

Between November 2016 and November 2021, twenty-eight patients were identified at the weekly multi-disciplinary lung cancer conference at Uppsala University Hospital and were prospectively included. Inclusion criteria were stage 3 or oligometastatic disease, defined as a maximum of five lesions, according to ^18^F-FDG PET/CT performed in the routine clinical work-up. Exclusion criteria were general state of health not permitting CRT (performance status ECOG-WHO > 2) or contra-indications for contrast-enhanced MRI, such as claustrophobia, pacemaker or severe renal insufficiency.

### Image acquisition

A WB-MRI (Achieva 1.5T, Philips) was performed with a total scan time of approximately 45 min. The MR protocol of the thorax and abdomen consisted of transaxial Dixon sequences including water, fat, in-phase and out-of-phase series (acquired voxel size 1.30 mm right-left (RL) x 1.70 mm anterior-posterior (AP) with slice thickness 5 mm feet-head (FH), field of view FOV 375 mm (RL) x 301 mm (AP) x 240 mm (FH), NSA 1, TR 5.50 ms, TE1 1.73 ms, TE2 3.70 ms, scan time 17 s) and DWI sequences with b-values 0, 50, 800 and a ADC map (acquired voxel size 3 mm (RL) x 3 mm (AP) with slice thickness 5 mm (FH), FOV 375 mm (RL) x 301 mm (AP) x 240 mm (FH), NSA 1, TE 93.31 ms, TR 2226.26 ms, scan time 3 min, 35 s.

The spine was examined from the skull base to coccyx with two sagittal T1 weighted (w) sequences, cervical and upper thoracic spine (acquired voxel size 0.89 mm (AP) x 1.25 mm (FH) slice thickness 3.00 mm (RL), FOV 160 mm (AP) x 347 mm (FH) x 62 mm (RL), NSA 1.5, TE 8 ms, TR 414.26 ms, scan time 2 min, 38 s) and lower thoracic and lumbar spine, sacrum and coccyx (acquired voxel size 0.89 mm (AP) x 1.25 mm (FH) with slice thickness 4 mm (RL), FOV 160 mm (AP) x 422 mm (FH) x 83 mm (RL), NSA 1.5, TE 8 ms, TR 414.26 ms, scan time 2 min, 41 s). The pelvis was examined with a coronal T1w sequence (acquired voxel size 1.76 mm (AP) x 1.76 mm (FH) with slice thickness 7 mm (AP), FOV 183 mm (AP) x 400 mm (FH) x 420 mm (RL), NSA 1, TE 12 ms, TR 590 ms, scan time 2 min, 30 s).

An extra cellular contrast agent (Dotarem, Guerbet) 279.3 mg/mL dosing 0.2 ml/kg body-weight with a maximum volume of 20 ml was administered with a power injector intravenously in an antecubital vein with a flow rate of 2 ml/s. The liver was scanned with a transaxial 3D T1w sequence (acquired voxel size 2 mm (AP) x 2 mm (RL) with slice thickness 4 mm (FH), FOV 256 mm (AP) x 400 mm (RL) x 210 mm (FH), NSA 1, TE 1.84 ms, TR 3.90 ms, scan time 13.4 s) in multiple phases (pre-contrast, 1 min, 3 min and 6 min post injection).

Finally, the brain was examined with a sagittal 3D T1w sequence (acquired voxel size 1.05 mm (AP) x 1.05 mm (FH) with slice thickness 1.1 mm (RL), FOV 240 mm (AP) x 256 mm (FH) x 160 mm (RL), NSA 1, TE 3.38 ms, TR 7.39 ms, scan time 5 min, 8 s) approximately 10 min after contrast administration.

The PET examinations were performed on non-digital (Discovery VCT, Discovery ST) or digital (Discovery MI) (GE-Healthcare) PET/CT-scanners. Examinations were performed according to clinical routine, with injection of 18 F-FDG 1 h prior to the scan start. Patients scanned on non-digital PET/CT received a dose of 4 MBq/kg using a 3 min/bed acquisition time. The patients scanned on the digital PET/CT were injected with 3 MBq/kg and underwent scanning with 2 min/bed.

### Image interpretation

WB-MRI was first reviewed by two readers separately, one (HH) with basic radiological training and one senior consultant radiologist (TH) with > 15 years of experience. Both were blinded for all previous imaging and clinical information, except that the patients suffered from lung cancer stage 3, oligometastatic disease or had undergone resection or CRT of the primary tumor.

The evaluated lesion characteristics included morphology (shape regular/irregular, delineation sharp/diffuse), contrast-enhancement (enhancement high/low, pattern homogenous/heterogenous) and diffusion restriction, defined as increased signal in b = 800 as compared to b = 0 and b = 50 and with corresponding decreased signal on the ADC (apparent diffusion coefficient) map.

Reviewer confidence for each lesion was assessed according to a five-point scale; 0 = not depicted (regarding primary tumor); 1 = low suspicion of malignancy; 2 = moderate suspicion of malignancy; 3 = strong suspicion of malignancy; and 4 = definite malignant tumor. Lesions that scored ≥ 2 were considered malignant. The grades 0 and 1 were not used in lymph node lesion grading in any of the modalities, as benign lymph nodes are present in healthy individuals and the exact total number was not of interest to this study.

Minor discrepancies between the reader’s results were resolved by consensus reading in a second joint session.

As reference standard, all available imaging as well as clinical information obtained at least 12 months after baseline were used, with exception for five patients who succumbed in shorter time than 12 months. This included all tomographic and nuclear medicine imaging available as well as histopathological results such as post-surgical pathological analysis, endo-bronchial ultrasound (EBUS) and other biopsy tissue samples from suspicious lesions. This was done for each specific lesion, potentially benign or malignant, detected in the WB-MRI and the clinically performed PET/CT examinations. Thus, each lesion depicted in the study examinations was evaluated, scored and determined as either benign or malignant and, in the subsequent comparison with the standard of reference, was noted as true positive, false positive, false negative or true negative. New lesions in other locations were not taken into account.

### Statistical analysis

R, version 3.1.6 (R Foundation for Statistical Computing, Vienna, Austria) was used for statistical analysis. All tests were performed for primary tumor, lymph node metastases and distant metastases separately. Lesions detected by ^18^F-FDG PET/CT and by WB-MRI were then patient-wise compared with the reference standard for primary tumor, lymph node metastases and distant metastases separately. The sensitivity and specificity of ^18^F-FDG PET/CT and WB-MRI were calculated using Clopper-Pearson. Finally, Fisher’s exact test was performed to compare the estimated sensitivity and specificity of ^18^F-FDG PET/CT and WB-MRI. *P* < 0.05 was considered significant.

## Results

All 28 patients successfully underwent ^18^F-FDG PET/CT and WB-MRI. WB-MRI was performed median 20 days (range: 0–37 days) after ^18^F-FDG PET/CT. In one patient, the presence of image artefacts in the central parts of the thorax in the DWI sequences somewhat complicated the image analysis. The study population consisted of 19 women and 9 men, with a mean age of 70.5 years (SD = 8.4) at study inclusion. The patients’ characteristics are described in Table 1.

**Table 1:** Study population characteristics

Patient number	Sex (F/M)	Age at diagnosis (years)	Histopathological result of tumor biopsy	Location of primary tumor (lobe)	Size of primary tumor (mm)	TNM stage
1	F	73	Adenocarcinoma	RUL	45 × 55	T4N2M0
2	M	57	Squamous cell carcinoma	RUL	57 × 26	T4N2M0
3	F	78	Poorly differentiated carcinoma	RUL	50 × 50	T2N2M0
4	M	80	Squamous cell carcinoma	RLL	100 × 60	T4N2M0
5	F	78	Adenocarcinoma	LUL	39 × 26	T2N2M0
6	F	74	Adenocarcinoma	LUL	*	rTxN1M0
7	M	55	Large cell carcinoma	LUL	80 × 80	T4N1M0
8	F	71	Adenocarcinoma	LUL	40 × 28	T2N3M0
9	M	72	Adenocarcinoma	LUL	21 × 22	T4N3M0
10	M	71	Squamous cell carcinoma	RUL	49 × 51	T2N1M0
11	F	75	Adenocarcinoma	RUL	20 × 19	T1N1M0
12	F	62	Adenocarcinoma	RUL	21 × 16	T3N0M1
13	F	77	Adenocarcinom	LUL	*	rTxN0M1
14	F	63	Squamous cell carcinoma	RUL	40 × 37	T4N2M0
15	F	78	Adenocarcinoma	ML	*	rTxN2M1
16	F	79	Poorly differentiated carcinoma	LLL	55 × 52	T3N1M0
17	M	74	Adenocarcinoma	RLL	23 × 11	T1N2M1
18	F	76	Adenocarcinoma	LUL	54 × 46	T4N0M0
19	F	55	Adenocarcinoma	RLL	-	rTxN2M0
20	F	74	Squamous cell carcinoma	LUL	57 × 37	T3N3M0
21	M	81	Squamous cell carcinoma	LUL	125 × 124	T4N3M0
22	F	55	Adenocarcinoma	LLL	*	rTxN0M1
23	F	54	Adenocarcinoma	RUL	-	rTxN3M1
24	F	74	Adenocarcinoma	LLL	73 × 43	T4N0M0
25	M	77	Adenocarcinoma	RLL	(-) 47 × 34	rT3N2M0
26	F	66	Squamous cell carcinoma	RLL	47 × 28	T3N2M1
27	F	76	Adenocarcinoma	LUL	53 × 20	T4N0M0
28	M	70	Adenocarcinoma	RLL	66 × 46	T4N2M0

Abbreviations: F, female; M, male; TNM, tumor-nodes-metastases (according to 8th edition of lung cancer classification); rTNM, recurrence or retreatment tumor-nodes-metastases (according to 8th edition of lung cancer classification); RUL, right upper lobe; RLL, right lower lobe; LUL, left upper lobe; ML, middle lobe; LLL, left lower lobe; *, resected; -, post radiochemo and/or immunotherapy; Tx, no remaining primary tumor visible at location of original tumor

Characteristics of patients (*n* = 28) including sex, patient age at time of diagnosis, histopathological tumor type, location and size of the primary tumor as well as TNM staging (8th edition) according to the multidisciplinary conference. In 7 cases, the patients had previously undergone treatment, either surgical resection (*n* = 4) or systemic therapy (*n* = 3), but developed recurrence in a different location than the original tumor. In those patients except one, no primary tumor could be measured in the TNM restaging (*n* = 6). In one patient (patient 25), the recurrence occurred in the location of the original tumor

### Primary tumor assessment

The scoring of the primary tumors is summarized in Table 2. There were 23/28 patients who on both modalities were harboring malignant primary tumors (score 2–4). On ^18^F-FDG PET/CT, 20/23 lesions were considered definitive malignant tumors (score 4) as compared to 18 lesions in WB-MRI. Follow-up used as standard of reference confirmed a malignant primary tumor in the reported 23 depicted lesions of the included 28 patients. In 5/28 patients, no primary tumor was depicted, neither by ^18^F-FDG PET/CT nor by WB-MRI. In these patients, surgery or successful CRT of the primary tumor had been performed. A total of 7 patients had undergone previous treatment but presented with relapse locally or with metastatic disease.

**Table 2:** Image interpretation and grading of primary tumor

Patient			WB MRI					PET CT			Reference standard
	0	1	2	3	4	0	1	2	3	4	
1					1					1	malignant
2					1					1	malignant
3					1					1	malignant
4					1					1	malignant
5					1				1		malignant
6	1					1					not depicted
7					1					1	malignant
8					1					1	malignant
9				1						1	malignant
10					1					1	malignant
11				1						1	malignant
12					1					1	malignant
13	1					1					not depicted
14					1					1	malignant
15	1					1					not depicted
16					1					1	malignant
17			1							1	malignant
18					1					1	malignant
19			1					1			malignant
20					1					1	malignant
21					1					1	malignant
22	1					1					not depicted
23	1					1					not depicted
24					1					1	malignant
25			1						1		malignant
26					1					1	malignant
27					1					1	malignant
28					1					1	malignant

Abbreviations: WB-MRI, whole-body magnetic resonance imaging; PET/CT, positron emission tomography computed tomography

Number of lesions of each grading score, 0–4. Grading of each lesion was performed for WB-MRI and ^18^F-FDG PET/CT respectively, according to a five-point scale and interpreted as 0 = not depicted; 1 = low suspicion; 2 = moderate suspicion; 3 = strong suspicion; and 4 = definite tumor. Lesions scoring 2 points or higher were considered malignant

### Assessment of thoracic lymph nodes

The number of thoracic lymph nodes and their respective scores in each patient, are summarized in Table 3. According to the reference standard, 17/28 (61%) patients harbored thoracic lymph node metastases. These were depicted on ^18^F-FDG PET/CT in all 17 patients (100%) and on WB-MRI in 11/17 (65%) patients. False positive findings of lymph node metastases occurred in 3 patients on ^18^F-FDG PET/CT and in 1 patient on WB-MRI.

**Table 3:** Image interpretation and grading of thoracic lymph node metastases

Patient			WB MRI					PET CT			Reference standard
	**0**	**1**	**2**	**3**	**4**	**0**	**1**	**2**	**3**	**4**	
1					1					1	1
2				1						1	1
3					1					1	1
4										2	2
5										1	1
6										1	1
7									2		2
8					7					12	12
9					3				1	3	4
10			1							2	1
11											0
12					9					11	11
13											0
14										3	0
15					3					4	4
16					5					12	12
17										1	1
18											0
19											0
20					6					6	6
21										1	1
22											0
23											0
24											0
25					2					2	2
26				1						5	0
27											0
28									1		0

Abbreviations: WB-MRI, whole-body magnetic resonance imaging; PET/CT, positron emission tomography computed tomography

Number of lymph node lesions of each grading score. Grading of each lesion was performed for WB-MRI and ^18^F-FDG PET/CT respectively, interpreted as 2 = moderate suspicion; 3 = strong suspicion; and 4 = definite tumor. Grading scores 0 = not depicted and 1 = low suspicion were not used in the setting of lymph nodes, as benign lymph nodes are expected findings and not of interest to this study. The reference standard column shows the number of lymph node metastases according to follow-up data

### Assessment of distant metastases

Table 4 summarizes the number of distant metastases, and their respective scores in each patient. According to the reference standard, 7/28 (25%) patients harbored distant metastases, some in multiple locations. In patients with oligometastatic disease, distant metastases were found in bone (7 patients), liver (5 patients), adrenals (5 patients) brain (4 patients), lung (3 patients), and pleura (1 patient). Both ^18^F-FDG PET/CT and WB-MRI depicted all distant metastases in all 7 patients. A false positive detection of distant metastases occurred in 3 patients on ^18^F-FDG PET/CT and in 5 patients on WB-MRI, which represents 21% (^18^F-FDG PET/CT) and 36% (WB-MRI) of the total number of suspicious distant metastatic lesions in both modalities.

**Table 4:** Image interpretation and lesion grading of distant metastases

Patient			WB MRI					PET CT			Reference standard	Location
	**0**	**1**	**2**	**3**	**4**	**0**	**1**	**2**	**3**	**4**		
1												
2												
3												
4		1					1				benign	liver
4			1								benign	bone
4							1				benign	lung
5				2			1				benign	lung
6			1						1		malignant	pleura
6				2						2	malignant	liver
7												
8												
9												
10												
11		1									benign	brain
11			1						1		benign	adrenal
12				1						2	malignant	bone
12					2						malignant	brain
13		1					1				benign	liver
13		1							1		malignant	liver
13					1					1	malignant	adrenal
14												
15		1	1				1				benign	bone
15				1						1	malignant	liver
16					1					1	malignant	bone
16					4						malignant	brain
17												
18												
19									1		benign	adrenal
20										1	benign	lung
21			1								benign	adrenal
22					1					1	malignant	bone
23					2					2	malignant	adrenal
23					5						malignant	brain
24			1								benign	liver
25												
26		1					1				benign	bone
27		1					1				benign	bone
28												

Abbreviations: WB-MRI, whole-body magnetic resonance imaging; PET/CT, positron emission tomography computed tomography

Number of distant metastases of each grading score in ^18^F-FDG PET/CT and WB-MRI respectively. Grading of each lesion was performed for WB-MRI and ^18^F-FDG PET/CT respectively, and interpreted as 1: low suspicion; 2: moderate suspicion; 3: strong suspicion; and 4: definite tumor. Grading score 0: not depicted was not used in the setting of distant metastases evaluation. Lesions scoring 2 points or higher were considered malignant. The reference standard column shows the number of distant metastases according to at least 12 months follow-up data. In patients with distant lesions in more than one location, results are presented in several rows, one for each location. The brain is not included in ^18^F-FDG PET/CT

### Statistical analyses

For primary tumor diagnosis, the sensitivity of WB-MRI and ^18^F-FDG PET/CT was identical, 1.00 (95% CI: 0.84-1.00), *p* = 1.00), as was the specificity, 1.00 (95% CI: 0.48-1.00), *p* = 1.00. For detection of lymph node metastases, the sensitivity was 0.65 (95% CI: 0.38–0.86) for WB-MRI and 1.00 (95% CI: 0.80-1.00) for ^18^F-FDG PET/CT, *p* = 0.018, and the respective specificities were 0.91 (95% CI: 0.35–0.85) and 0.73 (95% CI: 0.63-1.00), *p* = 0.59. For detection of distant metastases, the sensitivity was the same for WB-MRI and ^18^F-FDG PET/CT 1.00 (95% CI: 0.79-1.00), *p* = 1.00, and the respective specificities were 0.76 (95% CI: 0.79-1.00) and 0.86 (95% CI: 0.81-1.00), *p* = 0.70.

## Discussion

The present study, aimed at evaluating the additional value of WB-MRI, as compared to standard clinical work up with ^18^F-FDG PET/CT and brain MRI, in patients with locally advanced or oligometastatic NSCLC, showed comparable sensitivity and specificity regarding T and M staging, but significantly less mediastinal lymph node metastases were detected by WB-MRI than ^18^F-FDG PET/CT. Thus, no added value was found for adding WB-MRI to the routine imaging work-up.

For detection of the primary tumor and distant metastases, our results are in line with those of a number of previous studies [16, 18] including the systematic review and meta-analysis by Machado Medeiros et al. [18], showing similar sensitivity and specificity for ^18^F-FDG PET/CT and WB-MRI. In contrast to one previous study [15] we found no superiority of WB-MRI to ^18^F-FDG PET/CT. However, the MRI protocol in the study by Takeneka et al. [15] was more extensive, including a larger number of MRI sequences and anatomical imaging planes. Their total scan time was 90 min as compared to 45 min in the present study.

For hilar and mediastinal lymph node metastases (N staging), our results differ from those of a number of previous studies, which showed higher specificity for MRI than for ^18^F-FDG PET/CT [12–14]. However, in these previous studies the WB-MRI protocol was more extended, including imaging of the thorax in several anatomical planes, and applying T1- and T2-weighted sequences and short inversion time inversion recovery (STIR) sequences.

A limitation of this study was the small study population of 28 patients, of whom only 7 harbored distant metastases. The statistical analysis was therefore limited in terms of generalizability. Further, an extracellular MRI contrast agent was administered, due to the need for detection of brain metastases in these advanced NSCLC patients. This could potentially have reduced the ability to visualize and characterizing liver lesions, as compared to using a hepatocyte specific contrast agent. Moreover, our choice of a somewhat limited WB-MRI protocol, in order to avoid extended examination times, could potentially have contributed to the inferiority of WB-MRI in the N-staging and non-superiority in T- and M-staging, as compared with ^18^F-FDG PET/CT.

## Conclusions

In conclusion, WB-MRI showed no added value, as compared to MRI of the brain only, together with clinical routine ^18^F-FDG PET/CT for staging in patients with advanced.

NSCLC. Our findings suggest that adding WB-MRI into clinical routine in stage 3 NSCLC patients would not improve the process of correct staging, which is important to establish considering WB-MRI being a more expensive and time-consuming examination compared to brain MRI only.

## Electronic supplementary material

Below is the link to the electronic supplementary material.

Supplementary Material 1

## Data Availability

Data is provided within the manuscript or supplementary information files.

## Abbreviations

NSCLC: Non-small cell lung cancer
WB-MRI: Whole body magnetic resonance imaging
^18^F-FDG PET/CT: ^18^F-fluorodeoxyglucose positron emission tomography computed tomography
CRT: Chemo-radio therapy
RL: Right-left
AP: Anterior-posterior
FH: Feet-head
FOV: Field of view
NSA: Number of signal averages/acquisitions
TE: Echo time
TR: Repetition time
STIR: Short inversion time inversion recovery
CI: Confidence interval
